# Psychological Distress and Health Insurance Coverage among Formerly Incarcerated Young Adults in the United States

**DOI:** 10.3934/publichealth.2015.3.227

**Published:** 2015-06-24

**Authors:** Larrell L. Wilkinson, Saundra H. Glover, Janice C. Probst, Bo Cai, Lisa T. Wigfall

**Affiliations:** 1 Department of Human Studies, School of Education, University of Alabama – Birmingham, 901 13^th^ Street South, EB 210A, Birmingham, AL 35294-1250, USA; 2 Institute for Partnerships to Eliminate Health Disparities, Arnold School of Public Health, University of South Carolina, 220 Stoneridge Drive Suite #208, Columbia, SC 29210, USA; 3 South Carolina Rural Health Research Center, Arnold School of Public Health, University of South Carolina, 220 Stoneridge Drive Suite #204, Columbia, SC 29210, USA; 4 Department of Epidemiology and Biostatistics, Arnold School of Public Health, University of South Carolina, 800 Sumter Street, Columbia, SC 29208, USA

**Keywords:** incarceration, psychological distress, young Adults, mental Health, health insurance

## Abstract

The United States incarcerates more people per capita than any other nation. Studies have consistently demonstrated higher prevalence of serious mental illness among the incarcerated. Although health care may be available to individuals while incarcerated, research is needed to understand the context of health care coverage and mental health after incarceration. The purpose of this study is to estimate the point prevalence of psychological distress (PD) among young adults with incarceration experience, while comparing the prevalence to that of young adults in the general population. Additionally, this study characterizes the relationship between incarceration experience and PD, while also examining this association given an individual's health insurance coverage status among young adults. Lastly, we examine if other individual, contextual, and behavioral factors influences the relationship between incarceration experience and PD, in addition to their health insurance coverage status. This study utilizes data from the 2008 panel of the National Longitudinal Survey of Youth 97, a population based survey dataset from the U.S. Department of Labor. Andersen's Behavioral Model of Health Services Use provided the conceptual framework for the study. The Mental Health Index 5 (MHI-5) was used to determine PD or normal mental health. Chi-square testing and multivariate logistic regression were performed to examine incarceration experience in association to PD. The sample with incarceration experience reported almost double the proportion of PD (21%) compared to those without an incarceration experience (11%). Young adults who have been incarcerated reported greater odds of PD than those with no incarceration experience (COR 2.18; 95% CI, 1.68–2.83) and the association was diminished in the presence of health insurance status and model covariates. Future health prevention and health management efforts should consider the impact of health insurance coverage status, health behaviors, and life satisfaction on mental health status among young adults with incarceration experience.

## 1. Introduction

The United States (U.S.) incarcerates more people per capita than any other nation and the prison population has increased fourfold during the past 25 years [1]. As of 2009, nearly 1% of U.S. adults received their healthcare from their jailers (nearly 2.3 million inmates). Each year, almost 700,000 mentally ill people are placed in U.S. jails [2]. Studies in Chicago, IL, have suggested that 9% of male inmates had a severe mental disorder and 6% had an episode prior to arrest [2]. Freudenberg highlights a 1998 Bureau of Justice report suggesting 16% of state prison inmates, 7% of federal inmates, and 16% of probationers reported either a mental condition or overnight stay in the mental hospital [2]. Historically, the Epidemiological Catchment Area Study (ECA) was one of the first studies to estimate the point prevalence of mental illness among this segment of the U.S. population. The study found that prevalence rates of current and lifetime major depression, mania, schizophrenia, and other severe disorders were significantly higher among the jailed sample than within the entire five-city non-institutionalized sample of the ECA, even when controlling for race [3]. Other studies that followed affirmed the high rates of mental illness and distress among adjudicated females in the United States [4]–[6] and men internationally [7],[8].

Prisoners in the U.S. have the right to health care, according to the Eighth Amendment. Still, there is little known about prisoners' healthcare access and the linkage to mental health [1]. Using data from surveys examining health care utilization among local, state, and federal inmates, Wilper and colleagues [1] found an estimated 14% of federal inmates, 20% of state inmates, and 68% of local jail inmates received no medical examination since incarceration. Interestingly, almost 15% of federal inmates, 26% of state inmates, and 25% of local jail inmates had at least 1 previously diagnosed mental condition and most had taken medication prior to being incarcerated [1]. Also, most inmates in the study were in poor health, reporting high rates of chronic medical conditions, and self-reported substance abuse disorders [1],[9]. In addition, federal reports detail increased rates of infectious disease (i.e. HIV/AIDS, Hepatitis C) among former inmates [10].

While accessing care is important among the incarcerated, researchers are also examining access to care among the formerly incarcerated. Once released, those previously incarcerated may experience increased risk of mortality due to drug overdoses, suicide, homicide, chronic conditions, and infectious diseases [11]–[13]. A study among the U.S. metropolitan area with the largest population of former prisoners, Los Angeles County, California, found formerly incarcerated individuals reported similar rates of trouble accessing medical care, health insurance coverage for 12 months, self-reported general health status; but higher proportions of depression when compared to the general population in Los Angeles County [14]. Among a sample of 324 formerly incarcerated respondents (235 males and 89 females) in Baltimore, Maryland, 30 percent reported wanting help in acquiring mental health treatment, one-fourth reported experiencing serious anxiety or depression, and one-fifth reported experiencing symptoms of Post-Traumatic Stress Disorder (PTSD) within three months of being released. However, only 10 percent reported private insurance coverage and less than 5 percent reported having a government based coverage option [15].

Having health insurance coverage has been linked to improvements in psychological health [16]. The Rand Health Insurance Experiment (HIE) estimated the effects of variation in cost sharing on the health status among randomly enrolled individuals and families from six U.S. city/county areas over a three year period beginning in November 1974. Findings from the HIE indicate individuals who had poorer psychological well-being (PWB) gained more improvement in PWB on the free plan (no cost sharing, no coinsurance, or individual deductible) than on plans with coinsurance [16]. Findings from the HIE regarding psychotherapy also indicate cost sharing reduced the probability of seeking mental health care, specifically within a given year [16]. During the 1990s, the HealthCare for Communities (HCC) study explored developments in health insurance coverage and perceived access to care for persons with mental illness compared to persons in the general population [17]. Focusing on participants who might have substance abuse or poorer financial status, results suggest participants with probable mental disorder were more likely to report having lost health insurance, to report health insurance as depreciated, and more likely to find that access to care had become more difficult. These individuals were also less likely to report gaining insurance, if they previously had no insurance coverage [17]. Individuals who reported these circumstances were more likely to be female, younger (age 34 or less), have lower income, and be less educated. When analyzing privately insured only, findings suggest individuals most at risk to suffer depressive disorder were also more likely to report trouble accessing health care when compared to those who were not likely sufferers of depressive disorder. Also, individuals who were privately insured with psychological distress (PD) were more likely to perceive their access to healthcare as more difficult than privately insured with normal mental health [17].

More recently, a study conducted by the National Center for Health Statistics (NCHS) of the Centers for Disease Control and Prevention (CDC) released findings from the 2001–2004 administrations of the National Health Interview Survey (NHIS). Using the Kessler 6 (K6) scale, the purpose of the study was to estimate the prevalence of serious psychological distress (SPD) among the noninstitutionalized adult population of the United States [18]. Results indicated greater proportions of SPD were found among adults who were: ages 18–44, female, had less than a high school diploma, living in poverty, unmarried, cigarette smokers, reporting chronic conditions, or reported utilizing more medical services. In addition, the study found that persons with SPD were four times more likely to have Medicaid than persons without SPD. Furthermore, adults with SPD were significantly more likely to be uninsured than adults without SPD [18].

According to the U.S. Department of Justice, in 1999 50% of individuals released from prison were 29 years of age or younger [19]. Although national findings detailing rates of mental disorders among the current incarcerated are more prevalent; little research examining psychological health in association with health care coverage after incarceration has been conducted, particularly among a younger segment of the population in the U.S., given previous reports suggesting health coverage is lower among younger adults [20]. Therefore, the purpose of this study is to estimate the point prevalence of psychological distress (PD) among young adults with incarceration experience, while comparing the prevalence to that of young adults with no incarceration experience. The odds of reporting PD given incarceration experience in comparison to no incarceration experience is also described. Additionally, this study characterizes the association of incarceration experience classification and PD given an individual's health insurance coverage status among young adults. Lastly, we examine if other individual, contextual, and behavioral factors influence the association of incarceration experience and PD, in addition to their health insurance coverage status among young adults.

## 2. Materials and Method

### 2.1. Study Population

We analyzed data from the 2008 panel of the National Longitudinal Survey of Youth 97 (NLSY97), a population-based survey conducted by the U. S. Department of Labor since 1997. The U.S. Department of Labor began the NLSY97 to assemble data on the transition from school to work and into adulthood, collecting information on start and stop dates of jobs, occupation, industry, hours, earnings, job search, and benefits. Measures of education, work experience, length with an employer, and employer transitions were also obtained. Other information collected in a self-report questionnaire includes: youths' relationships with parents, dating, sexual activity, criminal behavior, and alcohol and drug use [21]. The youths, who are now adults, continue to be interviewed on an annual basis.

#### Data Sources

In 2008, 7,490 participants took part in the NLSY97 follow-up interviews. At time of follow-up, participants were 23–29 years of age. Each round of interviews was conducted using a computer-assisted personal interview (CAPI) instrument, administered by an interviewer with a laptop computer. To ensure that accurate data were collected from Spanish-speaking respondents, both English and Spanish versions of all survey instruments were used, and bilingual Spanish-speaking interviewers were employed to administer the Spanish version to those requesting it. During the initial round, the Spanish version of the questionnaire was requested by 297 responding parents and 96 NLSY97 youths [22].

### 2.2. Study Variables

#### 2.2.1. Independent Variables

The key independent variable was having experienced incarceration. In the NLSY97, having incarceration experience may be assessed with the item entitled “After Incarceration: How hard to stay out of prison next five years?” The respondent is asked, *How easy or hard do you think it will be to stay out of prison for the next five years?* This item was asked only to participants who had been previously incarcerated, as persons asked this question were identified through previous survey questions during the panel interview or identified earlier during previous NLSY97 interviews. Therefore, we identified those not asked this question, as those with no incarceration experience.

Several questions determined if the respondent had full year, partial-year, or no health coverage during the past year. In order to ascertain full year, partial-year, or no health coverage during the past year, the following questions were examined in combination:

1) *Do you have any kind of health care coverage, including health insurance, prepaid plans such as HMOs, or government plans such as Medicaid?; 2) Since [date of last interview], was there any time that you did not have any health insurance or coverage?; 3) Since [date of last interview], was there any time that you had health coverage?*

The answer categories for these questions were dichotomous “Yes” and “No” responses. Question #2 was asked of participants whom answered “Yes” to question #1, while question #3 was asked of participants whom answered “No.” Yes responses to questions #2 and #3 were combined to form the partial-year category for health insurance coverage. In order to determine type of health coverage, the question concerning primary health or hospitalization plan was employed: *What is the source of your primary health or hospitalization plan? Is it from a policy from your current or previous employer, a policy bought directly from a medical insurance company, is it Medicaid or an alternative Medicaid provider, or is it from some other source?*

In accordance to U.S. census status, persons indicating their source of plan from the military, Veterans Health Administration, prison system, or Medicaid or Medicaid provider/Medi-Cal/Medical Assist/Welfare/Medical Service were categorized as receiving government based health insurance coverage. Excluding “supervisor review” and “uncodable”, the other sources of health insurance coverage were categorized as private health insurance coverage [20]. The questions were combined in SAS 9.2 to create the following categories: Completely Uninsured; Partial-Year Insured/Unknown Source; Partial-Year Insured/Public; Partial-Year Insured/Private; Full year Insured/Public; and Full year Insured/Private. Full year coverage with private insurance was set as the referent category for analysis.

#### 2.2.2. Explanatory Variables

The Behavioral Model of Health Services Use suggests that health services use and health outcomes, such as PD are affected by social and individual determinants of health [23]. In the model, the predisposing, enabling, and need characteristics at the community and individual levels predict personal health practices, including the use of health services, and ultimately, health status. Individual measures may include demographic information, health conditions, and individual beliefs. Contextual characteristics are measured at the aggregate level and may include community characteristics and organizational characteristics such as: urban versus rural residential status. Health behaviors may include seeking medical services when injured or ill, not seeking medical services when injured or ill, and substance use [23].

In the Behavioral Model of Health Services Use, *predisposing* characteristics describe the propensity of individuals to use health services such as routine medical checkups or explain personal health practices such as substance use. Predisposing factors include socio-demographic characteristics such as gender (Male, Female), age, race/ethnicity (White, Black, Hispanic, Mixed), marital status (Never Married, Married, Separated, Divorced, Widowed), education (below high school graduation, high school graduate, and greater than high school graduate), household size (1–5, or more), place of residence (Rural, Urban, or Unknown), and geographic region (West, South, Northeast, North Central, or Unknown) of the United States [24]. *Enabling* characteristics refer to the individual's ability to gain access to needed health services. Potential health care access issues are related to family resources such as insufficient household income (less than $20,000, $20,000–$34,999, $35,000–$54,999, $55,000–$74,999, $75,000 or more) and lack of adequate health insurance coverage [24]. Perceived *need* for care is also a component of this model. Need includes self-perception of health status (fair/poor vs. good & above), social satisfaction (1–5, 6–7, 8–9, 10 being the highest) and clinically diagnosed chronic conditions such as asthma, cardiovascular disease, and diabetes (yes or no) [24]. Self-perception of good health or overall social satisfaction may decrease perceived need for and subsequent use of health services. In contrast, patients with actual health needs are likely to have more health care encounters that may result in more opportunities to discuss other preventive health services with their health care provider [24]. Personal health practices are also described in the Behavioral Model of Health Services Use. Personal health practices include health behaviors such as using alcohol during work or school (yes or no), smoking cigarettes (smoker or non-smoker), and marijuana usage (user or non-user) [23].

### 2.3. Outcome (Dependent) Variables

The main outcome variable of interest was self-reported mental health status (PD) using the Mental Health Index 5 (MHI-5). The MHI–5 consists of 5 items from the full RAND 36-Item Short-Form Health Survey measuring mental wellbeing and mental distress [25],[26]. Previous studies have suggested the MHI-5 is both comparable to other brief psychological measures, and internally consistent (α = 0.84) [27]. The items of the MHI-5 measure risk for suffering anxiety and depression, loss of behavioral or emotional control, and overall psychological well-being [28]. The 5-item measure of mental health contains the following questions: “How much of the time during the last month have you: (i) been a very nervous person? (ii) felt downhearted and blue? (iii) felt calm and peaceful? (iv) felt so down in the dumps that nothing could cheer you up? and, (v) been a happy person?” [29].

Within the self-reported questionnaire of the NLSY97, the participants were asked to rate questions on a 4-point scale. Each of the five questions were scored from 1 to 4: all of the time (1), most of the time (2), a little of the time (4), or none of the time. Because items (iii) and (v) ask about positive feelings, their scoring was reversed [30]. The five questions were combined and then changed through simple linear transformation to produce a continuous scale from range 0–100. Higher scores along the continuum indicate healthier psychological well-being. Additionally, research suggests that the scale has a cut-point in order to create a dichotomous measure for PD. Previous research has used a score of 52 for this purpose, with scores 52 or lower indicating PD [26],[31].

### 2.4. Statistical Analysis

Weights were applied using data from the Bureau of Labor Statistics to account for survey design, response rates and to yield national estimates for the young adult population of the U.S. We used chi-square tests to examine young adult population characteristics by incarceration experience and then examined the young adult population characteristics by mental health status. We then used logistic regression models to assess the bivariate association of incarceration experience and PD; incarceration experience and PD with health insurance status; incarceration experience and PD with health insurance and demographic variables; and incarceration experience and PD with predisposing, enabling, need, and access variables in addition to health insurance coverage. Covariates were selected based on a review of the scientific literature and variables that were available from the 2008 NLSY97 data. To obtain “better fit” models, covariates were added to the regression models controlling for basic demographic factors (age, gender, race, income, and education) and factors that differed significantly by incarceration experience and mental health status groupings. For all analyses, SAS version 9.2 (SAS Institute Inc., Cary, NC) was used. Analysis of the secondary data set received IRB approval (exempt status) from the University of South Carolina Internal Review Board on January 25, 2011.

## 3. Results

In the 2008 panel of the NLSY97, 498 persons were formerly incarcerated. Those with incarceration experience were comprised of 405 (79.85%) males and 93 (20.15%) females. The participants surveyed with incarceration experience were 61.01% (205) Non-black/non-Hispanic (White), 22.88% (173) Black, 14.67% (115) Hispanic, and 1.44% (5) mixed. Over half of participants surveyed with incarceration experience had less than a high school degree. Almost 76% of those with incarceration experience had never been married (Table 1). Also of interest, approximately 57% of the formerly incarcerated were completely uninsured, when compared to slightly over 20% of those with no incarceration experience (Table 1). Additionally, greater proportions of those with incarceration experience reported PD (20.73%), compared to those with no incarceration experience (10.72%). Significant differences were also observed within self-reported life satisfaction, whereas 29.84% of those with incarceration experience reported life satisfaction at the lowest levels, versus those with no incarceration experience (12.32%) (Table 1).

**Table 1.** Weighted chi-square analysis of the characteristics of the young adult population ages 23–29 by incarceration experience, National Longitudinal Survey of Youth 97.

Covariates	Inc. Exp.		No Inc.Exp.		*p*-value
	n ^b^	% ^c^	n ^b^	% ^c^	α = 0.05
**Age**					< 0.0001 ^a^
23–25	180	34.73	2948	41.23	
26–29	318	65.27	4044	58.77	
**Gender**					< 0.0001 ^a^
Male	405	79.85	3362	49.36	
Female	93	20.15	3630	50.64	
**Race and Ethnicity**					< 0.0001 ^a^
White	205	61.01	3592	71.06	
Black	173	22.88	1853	14.92	
Hispanic	115	14.67	1480	12.73	
Mixed	5	1.44	67	1.29	
**Education**					< 0.0001 ^a^
< High School	288	55.34	1298	16.21	
= High School	118	26.51	1807	25.06	
> High School	86	18.16	3822	58.73	
**Income**					< 0.0001 ^a^
< 20,000	175	30.93	703	14.85	
20,000–34,999	77	16.48	986	13.93	
35,000–54,999	73	16.50	1221	17.56	
55,000–74,999	38	8.79	886	8.79	
75,000+	48	10.89	1713	26.68	
Unknown	87	16.40	978	13.75	
**Marital Status**					< 0.0001 ^a^
Never Married	390	75.98	4638	63.59	
Married	66	14.74	1960	30.68	
Separated	9	1.65	77	1.07	
Divorced	30	7.34	295	4.61	
Widowed	1	0.29	4	0.05	
**Household Size**					< 0.0001 ^a^
1	98	17.37	814	12.08	
2	101	20.53	1886	29.72	
3	117	24.70	1719	25.30	
4	88	19.01	1302	17.82	
5 or more	94	18.40	1270	15.08	
**Place of Residence**					< 0.0001 ^a^
Rural	108	25.29	1212	19.45	
Urban	375	71.99	5390	74.77	
Unknown	15	2.72	390	5.78	
**Region of Residence in United States**					< 0.0001 ^a^
West	107	20.81	1581	21.92	
South	217	40.21	2802	36.68	
Northeast	52	11.60	1110	16.62	
North Central	118	26.95	1446	24.01	
Unknown	4	0.43	53	0.78	
**Health Factors**					
**Alcohol Usage Before and During Work**					< 0.0001 ^a^
None	429	87.40	6432	93.14	
User	69	12.60	560	6.86	
**Smoke Cigarettes**					< 0.0001 ^a^
Non-Smoker	193	34.91	4690	65.03	
Smoker	305	65.09	2302	34.97	
**Marijuana Usage**					< 0.0001 ^a^
None	379	76.28	6088	86.53	
User	119	23.72	904	13.47	
**General Health**					< 0.0001 ^a^
Good and Above	428	85.48	6382	92.25	
Fair/Poor	70	14.52	606	7.75	
**Life Satisfaction Score (1 = Least Satisfied)**					< 0.0001 ^a^
1–5	146	29.84	905	12.32	
6–7	133	28.80	1793	25.49	
8–9	136	27.59	3076	46.17	
10	80	13.77	1205	16.02	
**Chronic Disease**					< 0.0001 ^a^
None	461	93.37	6634	94.92	
One or More	37	6.63	355	5.08	
**Check-up during the past year**					< 0.0001 ^a^
No	325	69.57	3294	49.09	
Yes	173	30.43	3687	50.91	
**Received Treatment in Past Year when ill or injured**					< 0.0001^a^
None	351	68.41	4436	61.43	
1 time	77	17.08	1304	19.15	
2 times	28	4.98	622	9.56	
3 times	18	3.98	266	4.31	
4 or more times	22	5.56	353	5.56	
**Received No Treatment in Past Year when ill or injured**					< 0.0001 ^a^
None	344	67.27	4158	58.09	
1 time	51	10.36	975	14.06	
2 times	49	11.51	868	13.08	
3 times	25	5.77	461	6.92	
4 or more times	24	5.09	502	7.85	
**Variables of Interest**					
**Health Insurance Coverage Type by Year**					< 0.0001 ^a^
**Full-Year | Private**	71	16.85	3463	53.52	
Full-Year | Government	59	11.13	675	7.84	
Partial-Year | Private	15	3.58	510	7.85	
Partial-Year | Government	11	1.98	152	2.02	
Partial-Year | Unknown	48	9.31	597	8.28	
Full-Year | Uninsured	293	57.15	1582	20.48	
**Psychological Health**					< 0.0001 ^a^
Distressed	95	20.73	738	10.72	
Normal	380	79.27	5915	89.28	

^a^ Indicates significant differences

^b^ Indicates non-weighted data

^c^ indicates weighted proportions data

An estimated 11.34% of young adults self-reported PD. Of those reporting PD, approximately 57% were female and almost 58% were between the ages of 26 and 29. Within those reporting PD, almost 68% were Non-Hispanic White, approximately 41% had greater than high school education, but approximately 22% earned less than $20,000.00 household income per year (Table 2). Approximately 77% of young adults reporting PD lived in urban areas. A sizable proportion reporting PD lived in the South (39%), reported smoking (52%), but almost 79% reported good or above overall health (Table 2). Regarding health care, roughly half reported a check-up during the past year and 53% reported not seeking medical treatment when injured or ill during the past year. An estimated 47% reporting PD also reported lowest levels of life satisfaction. The uninsured rate for this population was approximately 28% (Table 2).

**Table 2:** Weighted chi-square analysis of the characteristics of the young adult population age 23–29 by mental health status, National Longitudinal Survey of Youth 97

Covariates	(PD) Psyc. Distress		Normal Mental Health		*p*-value
	n ^b^	% ^c^	n ^b^	% ^c^	α = 0.05
**All**	833	11.34	6,295	88.66	n/a
**Gender**					< 0.0001 ^a^
Male	346	42.71	3,212	51.97	
Female	487	57.29	3,083	48.03	
**Age**					< 0.0001 ^a^
23–25	363	42.18	2,621	40.74	
26–29	470	57.82	3,674	59.26	
**Race and Ethnicity**					< 0.0001 ^a^
White	395	67.71	3,294	71.72	
Black	248	17.93	1,625	14.46	
Hispanic	181	12.95	1,315	12.52	
Mixed	9	1.41	61	1.30	
**Education**					< 0.0001 ^a^
< High School	266	30.29	1,206	16.77	
= High School	233	28.45	1,581	24.34	
> High School	325	41.26	3,454	58.89	
**Income**					< 0.0001 ^a^
< 20,000	209	22.44	1,086	14.68	
20,000–34,999	121	14.77	902	14.11	
35,000–54,999	129	16.35	1,119	17.90	
55,000–74,999	88	10.35	805	13.50	
75,000+	156	20.90	1,551	26.73	
Unknown	130	15.19	832	13.07	
**Marital Status**					< 0.0001 ^a^
Never Married	594	68.61	4,163	63.38	
Married	171	22.07	1,791	31.17	
Separated	21	2.81	62	0.89	
Divorced	44	6.32	260	4.51	
Widowed	1	0.19	4	0.06	
**Household Size**					< 0.0001 ^a^
1	111	13.32	764	12.34	
2	189	25.12	1,712	29.84	
3	187	22.15	1,548	25.53	
4	149	17.55	1,173	17.91	
5 or more	197	21.86	1,097	14.38	
**Place of Residence**					< 0.0001 ^a^
Rural	138	18.49	1,116	19.94	
Urban	661	77.47	4,815	74.11	
Unknown	34	4.04	364	5.96	
**Region of Residence in United States**					< 0.0001 ^a^
West	169	19.39	1,440	22.22	
South	348	39.18	2,507	36.32	
Northeast	133	16.66	970	16.24	
North Central	178	24.33	1,327	24.41	
Unknown	5	0.44	51	0.81	
**Health Factors**					
**Alcohol Usage Before and During Work**					< 0.0001 ^a^
None	737	89.66	5,813	93.48	
User	96	10.34	482	6.52	
**Smoke Cigarettes**					< 0.0001 ^a^
Non-Smoker	422	48.07	4,191	64.70	
Smoker	411	51.93	2,104	35.30	
**Marijuana Usage**					< 0.0001 ^a^
None	647	77.69	5,495	86.80	
User	186	22.31	800	13.20	
**General Health**					< 0.0001 ^a^
Good and Above	654	78.97	5,851	93.76	
Fair/Poor	178	21.03	443	6.24	
**Life Satisfaction Score (1 = Least Satisfied)**					< 0.0001 ^a^
1–5	373	46.65	620	9.03	
6–7	255	29.75	1,562	24.90	
8–9	140	17.71	2,965	49.20	
10	61	5.88	1,142	16.88	
**Chronic Disease**					< 0.0001 ^a^
None	794	95.72	5,964	94.84	
One or More	38	4.28	330	5.16	
**Check-up during the past year**					< 0.0001 ^a^
No	407	50.55	3,033	50.26	
Yes	425	49.45	3,255	49.74	
**Received Treatment in Past Year when ill or injured**					< 0.0001 ^a^
None	445	50.57	4,088	62.96	
1 time	153	19.18	1,176	19.16	
2 times	91	11.39	541	9.24	
3 times	39	5.20	228	4.10	
4 or more times	101	13.67	256	4.54	
**Received No Treatment in Past Year when ill or injured**					< 0.0001 ^a^
None	458	53.25	3,800	58.94	
1 time	103	11.96	885	14.17	
2 times	96	13.06	785	13.11	
3 times	63	7.67	412	6.89	
4 or more times	105	14.07	397	6.89	
**Variables of Interest**					
**Health Insurance Coverage Type by Year**					< 0.0001 ^a^
Full-Year | Private	261	34.61	3,153	54.08	
Full-Year | Government	142	15.55	543	6.93	
Partial-Year | Private	49	6.48	455	7.77	
Partial-Year | Government	32	3.30	117	1.76	
Partial-Year | Unknown	100	11.94	518	7.88	
Full-Year | Uninsured	248	28.12	1,501	21.58	
**Incarceration Experience**					< 0.0001 ^a^
Yes	95	11.34	380	5.55	
No	738	88.66	5,915	94.45	

^a^ Indicates significant differences

^b^ Indicates non-weighted data

^c^ indicates weighted proportions data

In the crude analysis, young adults who had been previously incarcerated reported greater odds of PD than those with no incarceration experience (COR 2.18; 95% CI, 1.68–2.83) (Table 3). When introducing health insurance coverage status in the second model; the odds of reporting PD were reduced (AOR 1.73; 95% CI, 1.31–2.28) (Table 3). Interestingly, partial-year private coverage was the only insurance coverage classification not significant within the analysis (Table 3). In the third model (Model 2 plus Demographics), PD and incarceration experience remains significantly associated. Significant covariates in the model include all health insurance categories except partial-year private coverage, gender (AOR 1.54; 95% CI, 1.29–1.84), less than a high school graduation (AOR 1.26; 95% CI, 1.01–1.58), and greater than high school graduation (AOR 0.69; 95% CI, 0.56–0.86) (Table 3). In the final analysis (Model 4) adjusted for Andersen covariates, the PD and incarceration experience is no longer significantly associated. Significant covariates in the model include gender (AOR 1.54; 95% CI, 1.23–1.85), marijuana usage (AOR 1.34; 95% CI, 1.06–1.70), reporting fair or poor health (AOR 1.54; 95% CI, 1.17–2.03), receiving treatment 4 or more times in the past year when ill or injured (AOR 1.79; 95% CI, 1.25–2.56), lower life satisfaction reporting (1–5) (AOR 12.45; 95% CI, 8.62–18.25) and life satisfaction reporting (6–7) (AOR 3.36; 95% CI, 2.34–4.83) (Table 3).

**Table 3.** Weighted logistic regression models for psychological distress among young adults ages 23–29, National Longitudinal Survey of Youth 97.

Model *(referent = no incarceration experience)*	OR	95% CI
**Model 1 Formerly Incarcerated**		
*Yes*	2.18	(1.68–2.83) ^a^
*No*	1.00	Referent
		
**Model 2 Incarceration Experience x Health Insurance Status**		
Formerly Incarcerated		
*Yes*	1.73	(1.31–2.28) ^a^
*No*	1.00	Referent
Health Insurance Status		
*Full-Year* | *Private*	1.00	Referent
*Full-Year* | *Government*	3.37	(2.62–4.33) ^a^
*Partial-Year* | *Private*	1.29	(0.92–1.83)
*Partial-Year* | *Government*	2.86	(1.79–4.57) ^a^
*Partial-Year* | *Unknown*	2.30	(1.75–3.03) ^a^
*Full-Year* | *Uninsured*	1.86	(1.51–2.30) ^a^
		
Model 3 Incarceration Experience x Health Insurance x Demographics**		
Formerly Incarcerated		
*Yes*	1.70	(1.27–2.27) ^a^
*No*	1.00	Referent
Health Insurance Status		
*Full-Year* | *Private*	1.00	Referent
*Full-Year* | *Government*	2.24	(1.67–3.02) ^a^

*Partial-Year* | *Private*	1.21	(0.85–1.73)
*Partial-Year* | *Government*	1.84	(1.09–3.13) ^a^
*Partial-Year* | *Unknown*	1.99	(1.49–2.66) ^a^
*Full-Year* | *Uninsured*	1.54	(1.21–1.95) ^a^
Significant Demographic Covariates		
Gender		
*Female*	1.54	(1.29–1.84) ^a^
*Male*	1.00	Referent
Education		
< *High School*	1.26	(1.01–1.58) ^a^
= *High School*	1.00	Referent
> *High School*	0.69	(0.56–0.86) ^a^
		
Model 4 Incarceration Experience x Health Insurance x Andersen Model Covariates***		
Formerly Incarcerated		
*Yes*	1.30	(0.94–1.80)
*No*	1.00	Referent
Health Insurance Status		
*Full-Year* | *Private*	1.00	Referent
*Full-Year* | *Government*	1.32	(0.94–1.85)
*Partial-Year* | *Private*	1.00	(0.68–1.47)
*Partial-Year* | *Government*	0.83	(0.44–1.59)
*Partial-Year* | *Unknown*	1.33	(0.96–1.84)
*Full-Year* | *Uninsured*	1.01	(0.77–1.33)
Significant Andersen Covariates		
Gender		
*Female*	1.50	(1.23–1.85) ^a^
*Male*	1.00	Referent
Marijuana Usage		
*Yes*	1.34	(1.06–1.70) ^a^
*No*		
General Health		
Good and Above	1.00	Referent
Fair/Poor	1.54	(1.17–2.03) ^a^
Received Treatment in Past Year when ill or injured		
None	1.00	Referent
*1 time*	1.15	(0.90–1.47)
*2 times*	1.24	(0.91–1.69)
*3 times*	1.08	(0.68–1.72)
*4 or more times*	1.79	(1.25–2.56) ^a^
Life Satisfaction Score (1 = Least Satisfied)		
*1–5*	12.45	(8.62–18.25) ^a^
*6–7*	3.36	(2.34–4.83) ^a^
*8–9*	1.18	(0.81–1.72)
*10*	1.00	Referent

^a^ Indicates significant differences

** Demographic variables include age, gender, race/ethnicity, education, and income.

*** Andersen Model covariates includes age, gender, race/ethnicity, education, income, health insurance status, marital status, household size, place of residence, U.S. region, usage of alcohol during school or work, tobacco usage, marijuana usage, chronic disease, receiving treatment when ill or injured, not receiving treatment when ill or injured, and life satisfaction.

## 4. Discussion

A nationally representative data set of young adults in the United States was used to demonstrate the association of reporting PD when having experienced incarceration. Furthermore, an exploration of how one's health insurance status alone may confound the relationship of incarceration experience and PD was conducted. This relationship was also examined in the presence of demographic and other covariates. In this study, health insurance status is extended beyond having reported coverage (yes or no) during the past year, but also examines what type of health insurance individuals might have, and if they had coverage for the entire year. Findings from this study are in accord with earlier research showing that different types of health insurance are associated with poorer mental health [16]–[18]. Similarly, this study demonstrates the prevalence of PD is significantly greater in the formerly prisoned population versus the general population when examining young American adults [3]–[10]. Among young adults with incarceration experience, roughly 21% reported PD, greater proportions reported being uninsured for the full year and lower proportions reported having had a medical check-up during the past year. Additionally, roughly 67% of formerly incarcerated young adults reported not seeking medical treatment when injured or ill during the past year.

By using methodologies similar to Pratt et al. [18], we found that PD is associated with many individual and contextual characteristics, health behaviors, and accessing medical care among young adults. Although the population dataset in the CDC study used NHIS data from 2001–2004 and included adults of all ages, findings between this study and the previous study are similar. For example, both studies found greater proportions of reporting PD among the lower age groups (younger adults), females, individuals living in poverty, persons unmarried, smokers, individuals with chronic conditions, and persons who utilized more medical services. Contrasting the study by Pratt et al., PD was reported in greater proportions among young adults with greater than high school education and full-year private insurance coverage in the bivariate analysis of the present study.

When examining PD in association with incarceration, results affirmed individuals with incarceration experience reported greater odds of PD. Additionally, findings from the data indicate health insurance status confounds the association between incarceration status and PD. Findings suggest that government-based health insurance adds to the risk of reporting PD among the formerly incarcerated. Although diminished in the presence of demographic variables, the risk of reporting PD is still greatest among formerly incarcerated young adults with full-year government based coverage. However, in the presence of other covariates, PD is explained by female gender, 30 day marijuana usage, reporting fair or poor health, lower levels of life satisfaction, and receiving medical treatment when ill or injured 4 or more times during the past year.

Challenges such as finding jobs with employer-based health coverage or problems with Medicaid enrollment may hinder those with incarceration experience from receiving health insurance coverage [32] or perhaps may be associated with more distress due to trouble accessing medical services [17]. One study estimates that roughly 34–40 percent of released inmates will qualify for Medicaid [32], and perhaps those that receive Medicaid will still experience PD. However, the need for health care, the complexity of social situations, fragmented social and family networks, and mental health needs; may be barriers to many individuals with incarceration experience accessing health care coverage [32], thus widening the gap for the formerly incarcerated to access care [14],[15] and presenting opportunity for future research. In this study, receiving treatment 4 or more times when ill or injured was significantly associated with PD in the logistic regression analyis. This is of great concern, as providing health insurance coverage for this population is only the first step to improving the health among young adults with incaceration experience. Although this group can be served by Medicaid, especially with Medicaid's expansion under the Affordable Care Act (ACA) [33], individuals with incarceration experience have relied on safety net resources to meet their health needs, such as emergency rooms. The ACA promotes the adoption of patient centered medical homes and preventive care. Thus it will be important to integrate mental health care with physical health care to support the reintegration of the formerly incarcerated into the community [32].

Life satisfaction was rated lower in greater proportion among the young adults with incarceration experience than among those with no incarceration experience. These findings among young adults, specifically those with incarceration experience, support the use of frameworks that consider individual beliefs (need characteristics), as well as predisposing and enabling factors when examining self-reported health outcomes at the population level. In order to depict a comprehensive representation of one's total health and functioning, health care professionals and health systems must examine not only one's physical and mental health, but also assess one's social health. In this study, the social health indicator “life satisfaction” was significantly associated with mental health and incarceration experience. Similarly, self-reported general health status (global health assessment) was also significantly associated with mental health.

### Limitations of the Study

The results of this study should be viewed through a perspective of several limitations. This report is limited by the self-report nature of the NLSY97. First, recall bias may limit some of the responses due to solicitation of information for the past 30 days, over the past year, or questions pertaining to ever experiencing certain conditions or problems. Secondly, although the MHI-5 has been used in population-based surveys, the characterization of the cut-point to categorize PD and normal mental health is receiving additional examination. Although previous studies have used 52 as the cut-off point when using the MHI-5, one study has indicated the use of higher cut-off points including 54 and 74 for different clinical purposes [34], and 60 or 68 in another study of similar scope [35]. Still, it is important to note that these studies use different gold standards to compare the MHI-5 measure and both studies suggest more research is needed among different populations to determine a true standard cut-off point.

Another limitation of this study is the NLSY97 data used in this analysis did not ask questions about mental health knowledge, attitudes, beliefs, or behavioral intentions. This additional information would have been useful in performing data analysis and taking into consideration a factor such as stigma with mental illnesses, mental illness treatment, or social desirability. The results may also be limited by missing data. Of the 8,894 participants of the NLSY97 study, 1,494 (16.8%) of the study population is missing due to not being interviewed during 2008. For example, included in the missing data within the study population may be active military serving the U.S. overseas.

Due to the cross-sectional analysis of the data from 2008, the temporal sequence for causation PD among young adults cannot be determined. Furthermore, the direction of associations is uncertain as poorer health status may lead to loss of health insurance coverage or gain, while lack of health insurance may worsen one's health; subsequently influencing medical treatment utilization. Moreover, the covariates in our logistic regression analysis may inaptly reduce the crude association between incarceration and PD, and the relationship adjusted by health insurance status. For example, marijuana usage, lower life satisfaction, poorer self-report general health status, and greater treatment seeking when ill or injured may result from PD. In focus, finding a covariate within the data linked to incarceration status but not associated with PD, and then examining differences in the incarceration variable induced by that covariate to assess incarceration's relationship to PD; would allow for a more valid representation of the association [36]. Still, to address endogenous variables by finding an instrumental variable is difficult and the variation in the explanatory variable may not be enough to establish the effect on the dependent variable [36]. However, one study has demonstrated an increase of the protective effect of health insurance on mortality by using an instrumental variable to account for endogeneity bias [37], thus providing opportunity for additional research.

Despite these limitations, this study is innovative because it takes a population based approach to examining the mental health status of a defined group that is at risk for poorer mental health due to their stage and station in life. Although not generalizable to the entire U.S. population, one strength of the study is its focus on the young adult population. Many studies focus on wide age categories such as 18–64 or 25–44 years of age. This study specifically examines adults, ages 23–29 years old, an age group encompassing almost half of formerly incarcerated undergoing reentry [12]. Furthermore, the random selection of the original cohort and the weighting of the data, allow this study to make population based estimates of PD for young adults and for the segment of the young adult population whom have incarceration experience. Unlike other population based surveys, the NLSY97 data does not rely solely on telephone based interviews. The interview staff conducted interviews in person, over the phone or in other settings. This allows for more concrete information to be gathered and avoids selection bias by participants who may change location frequently or may only have a cell phone. Finally, examining health insurance status beyond a simple dichotomous variable provides for a more detailed account of how type and length of coverage may influence mental health status.

## 5. Conclusions

European studies and publications from the World Health Organization have well documented the prevalence rates of PD. Recent publications in the *Journal of International Health* and other government documents have reported SPD and PD in the United States. To date, few if any studies have focused on the young adult population of the United States, and less focusing on the impact of health insurance coverage status, especially in a population with incarceration experience. The proportion of Americans who have experienced incarceration has grown over the years. Still, little is known about the ability of young adults within this group to access health care given insurance status and their psychological well-being. This study offered a unique opportunity to estimate the rate of PD within this vulnerable sub-group and compare it to that of the general young adult population. Given the ongoing implementation of the ACA since 2010 and the age group in this study, implications for young adult enrollment may contribute to increasing health equity within this segment of the population [38]. Interventions directly targeting this group may have a significant impact on improving health outcomes for those with incarceration experience and their families, thus impacting the community.
